# 

**Published:** 1918-08

**Authors:** 


### DEATHS.

Dr. Una Howe Nasskarl, wife of Major Robert A. Nasskarl of the 111th sanitary train, Camp Bowie, Fort Worth, was killed when an automobile in which she was riding turned over and rolled into a ditch.

Mrs. Nasskarl left Foft Worth and was on her way to her father, Dr. Howe, of Atlanta, Texas. She planned to make her home with him while her husband was in France. Major Nasskarl left for an Atlantic port Monday.

Mrs. Nasskarl was pinned under the wreckage and her chest crushed.

Dr. C. O. Smith, of Ratcliff, was instantly killed when the motor car in which he was riding was wrecked on the Eastern Texas Railway between Lufkin and Ratcliff.

Dr. Walter P. Breach, a widely known eye, ear and nose specialist died recently in his home in Galveston following a stroke of apoplexy a short while before.

Dr. J. G. Ellis, a prominent physician of Denison, received a telegram July 12th, informing him of the death of his son, Captain J. G. Ellis, Jr., from effects of gunshot wounds received in France July 2. Captain Ellis organized an ambulance company composed of fifty-three Denison young men last July. They trained at Fort Clark and landed in France in March. They have been in active service three weeks, and on the firing line a longer time, according to a letter received recently from Captain Ellis.

No Apparent Change: Sapleigh: I say if I were to lose my mind, would I—ah—be aware of it myself, you know?

Doctor: You would not notice the difference, nor would any of your friends.—*Boston Transcript.*

### Old Jokes That We Have Met.

"Mirandy, fo' goodness sake, don't let dem chickens outer dis here yard. Shut dat gate."

"Wat fur, Aleck; day'll come home, won't dey?"

"Deed dey won't. Dey'll go home."

---

THE DOCTOR stood by the bedside and looked gravely down at the sick man. "I cannot hide from you the fact that you are very ill," said he. "Is there anyone you would like to see?"

"Yes," said the sufferer faintly.

"Who is it?"

"Another doctor."

---

AT a concert held in a certain town a soldier of the Black Watch occupied a seat in front of a private of an Irish regiment and his sweetheart. The latter was very much interested in the Highlander's uniform, and scanned the regimental badge on his cap and collar particularly. This badge is the figure and cross of St. Andrew, with the motto, *"Nemo me impune lacessit"* (No one annoys me with impunity).

"Phwat does that wroitin' mane, Patsy?" asked the girl.

"Phwy," replied Pat, "it's Latin, but I've forgotten the English av it. But in good ould Oirish it manes, 'Thread on the tail av me coat if ye dare!' "

---

An Irishman who was ill went to London to consult an eminent specialist.

The doctor having examined him, said: "I should like to know whether your family have been long-lived."

"Well, doctor, I'll just tell you how it is," replied the patient musingly. "My family are West of Ireland people, and the age of my ancestors depended entirely on the judges and juries who tried them!"—Tit-Bits.

---

Confession.—"I hear that my friend Brown, whom you have been treating for liver trouble, has died of stomach trouble."

"Don't you believe it, sir! Don't you believe it! When I treat a man for liver trouble, he dies of liver trouble."—*Ex.*

**SUNBURN**
**URTICARIA**
**PRICKLY HEAT**
**CHAFES**
**HYPERDROSIS**
**DERMATITIS**
**HIVES**
**ITCHING**

**Summer Skin Discomforts**

require the use of a specially adapted dusting and dressing powder.

**KORA-KONIA**

is soothing, healing, absorbent, protective, the ideal agent intended for professional use by physicians, surgeons, specialists, nurses—and endorsed by them.

Samples sent on request.

THE HOUSE OF MENNEN NEWARK, N. J.

"I HEAR that Tightly has had a relapse. I thought that Doctor Squills cured him."

"He did. Then he sent in his bill."

---

THE PHYSICIAN to whom the Irishman had applied for relief from a stomach ailment asked on the occasion of his last visit:

"Have you been drinking the very hot water an hour before meal, as I directed? If so, how do you fell now?"

"Doc," said the Celt, "I tried hard to do it, but I had to quit. I drank for 35 minutes and it made me feel like a balloon!"

---

PHYSICIAN (to Mrs. Colonel Blood of Kentucky)—How did your husband pass the night, Mrs. Blood?

MRS. BLOOD—He seemed quite comfortable, sir, and asked for water several times.

PHYSICIAN (with a grave look)—H'm—still flighty.

